# The association between steps per day and blood pressure in children

**DOI:** 10.1038/s41598-022-05497-0

**Published:** 2022-01-26

**Authors:** Aneta Weres, Joanna Baran, Ewelina Czenczek-Lewandowska, Justyna Leszczak, Artur Mazur

**Affiliations:** Medical College, Institute of Health Sciences, University of Rzeszow, Al. mjr. W Kopisto 2 a, 35-310 Rzeszów, Poland

**Keywords:** Health care, Medical research, Risk factors

## Abstract

Lower levels of physical activity are associated with an increased overall cardiometabolic risk, as well as the risk or being overweight. It is difficult to determine the optimal level of physical activity that protects the needs of children and young people. Studies on the required number of steps, as well as approximating the daily volume of physical activity, are gaining increasing credibility in research and practice. Researchers propose a "rule" of ≥ 11,500 steps per day, for children and teenagers of both sexes. The aim of the study was to assess whether 11,500 steps a day is sufficient to maintain normal blood pressure among children and adolescents. 1002 children and adolescents aged 4–15 were included in the study. To assess physical activity, measured in the number of steps per day, the Actigraph accelerometer wGT3X-BT was used. The normal number of steps was defined as greater than or equal to 11,500 steps per day for children and teenagers, for both girls and boys. In the entire study group, a significantly lower risk of hypertension was observed when the number of steps was normal (OR is 0.45 and the upper confidence limit for OR is 0.71). The study confirmed the beneficial protective role of physical activity against hypertension in older children and adolescents. However, it should be emphasized that no such relationship has been demonstrated in the case of preschool children. The presented norms of the number of steps should be promoted to the wider community to make prevention of cardiovascular diseases even more effective.

## Introduction

For many decades, hypertension was considered a health problem affecting adults and elderly people; however, its incidence in the child population has also increased in the modern world. Primary hypertension (PHT), which may already develop in children, has become a significant world-wide health problem as it constitutes one of the important risk factors for the development of cardiovascular diseases, such as myocardial infarction and stroke, atherosclerosis and chronic kidney disease in adulthood^1,2^.

One of the methods of PHT prevention is a sufficient level of physical activity which ensures the correct functioning of the cardio-vascular system and has been recognized by experts of the World Health Organization as a major factor in the prevention of diseases of civilization^3,4^. A level of physical activity of children and youth that is too low in relation to needs is a problem occurring in all developed countries. The effects of its deficiencies usually manifest themselves after many years, and due to many factors affecting the health and development of the child it cannot always be proved that insufficient physical activity is the main cause of emerging health problems. It is difficult to determine the optimal level of physical activity, i.e. that which protects the health of children and young people.

In order to improve energy balance, a person should increase daily energy expenditure above the typical sedentary level of 300 kcal by about 400 kcal in the performance of aerobic physical exertion lasting about 45–60 min a day^5,6^.

A sufficient level of physical activity is becoming an important element of hypertension therapy, including cases where there is a genetic predisposition toward hypertension. One of the mechanisms of the beneficial effect of physical exertion is microRNA modulation. This plays an important role in regulating gene expression, including of those genes responsible for hypertension. Changes in the cardiovascular system caused by the inflammatory mechanism are dependent on microRNA-155^7^.

Earlier research has already highlighted the link between an increased daily step count and a reduced incidence of diseases like diabetes and high blood pressure. Although this relationship in adults has been described in the literature, there are few publications discussing this problem in children^8^. It is important to start the prevention of cardiovascular diseases at an early age, because research shows that increased arterial pressure in childhood and adolescence may predispose many to hypertension in adulthood, thereby increasing cardiovascular risk. Childhood and adolescence is the best time to shape proper eating habits and a healthy lifestyle, including physical activity.

Studies on the required number of steps, as well as approximating the daily volume of physical activity, are gaining increasing credibility in research and practice. In 2004, Cardon estimated that the number of steps in school children corresponding to 60 min of moderate-to-vigorous exercise is 13,130 a day, and in preschool children it is 13,874 steps/day^9^. Rowlands estimated that the standard 60 min of moderate-to-vigorous physical activity (MVPA) is equivalent to on average 16,500 steps for children aged 8–10^10^. In a group of Japanese preschool children, Tanaka obtained a result of 9934 steps/day^11^. In 2011, Catrine Tudor-Locke et al. defined moderate activity as corresponding to between 10,000 and 12,000 steps per day^12^. Due to the fact that the studies have not agreed on a precise determination of a specific value for the number of steps in people under 18 years of age, in 2013, taking into account current data, Adams et al. proposed a "rule" of ≥ 11,500 steps per day, for children and teenagers of both sexes, assuming that the more steps taken daily, the better^13^.

Obtaining a clear conclusion on the effects of walking on blood pressure is fundamental to the development of new and more effective methods of preventing heart disease, which is a major health complication of high blood pressure.

Physical activity cannot be measured by one parameter alone, as it is a complex and multidimensional phenomenon. The large variety of methods used to assess physical activity causes difficulties in actual assessment of the scale of the problem in the world and indicates a need for research in this field.

Detailed recommendations on level and intensity required for health benefits are needed, especially with regard to age groups and sex.

The aim of the study was to assess whether 11,500 steps a day is sufficient to maintain normal blood pressure among children and adolescents.

## Materials and methods

### Participants

The study covered 1300 children attending randomly selected primary schools and kindergartens in the Podkarpackie Voivodeship in Poland. The criterion for inclusion in the study was consent of the parent or legal guardian and the child to participate in the study, age of the child between 3 and 18 years old. The parents were provided with information about the course of the research and the possibility of withdrawing at any stage. After written, informed consent for the child's participation in the study was obtained from the parents, 1196 children were included in the study.

In 74 subjects insufficient monitoring time was found on working days, in 86 subjects insufficient monitoring time was found on free days (Saturday–Sunday), and 34 subjects did not achieve a sufficient number of both working days and free days. Finally, 1002 children and adolescents aged 4–15 were included in the study. Personal data were secured and participants were given an appropriate digital code. The approval to conduct the study was obtained from the Bioethics Commission at the Medical Faculty of the University of Rzeszow No. 19/12/2015 of December 2, 2015. All methods were performed in accordance with the relevant guidelines and regulations by including a statement.

### Anthropometric measurements, blood pressure and physical activity

For measuring body height, a SECA 213 telescopic portable stadiometer was used (Seca Gmbh & co.kg Hammer Steindamm 9–25 22089 Hamburg, Germany CE 0123). The height measurement in this study is taken to the nearest 0.5 cm. Blood pressure was assessed by automatic oscillometric measurement using the Welch Allyn, Inc., 4200B-E2 (Skaneateles Falls, NY, USA) along with a set of cuffs of various widths, intended for both children and adults. These criteria have been approved by the Association for the Advancement of Medical Instrumentation (AAMI)^14^. The TANITA BC-420 MA analyser (with children ≤ 6 years old) and TANITA BC 418 MA (with children > 6 years old) were used to measure body weight, which meets all the requirements in accordance with European standards and directives. The analyser has a 3rd class medical device certificate and meets the EU standard for medical devices MDD 93/42 EEC. Based on the measurements of body weight and height, the body mass index (BMI) was determinedThis value has been classified based on the recommendations of the International Obesity Task Force (IOTF)^15^.

Blood pressure was classified using percentile grids of arterial pressure values for children aged 3–6 years and 7–18 years, by sex and age as well as by sex and height, developed on the basis of the OLA and OLAF projects (developing a blood pressure standard for children and adolescents in Poland) and based on oscillometric measurements^16^. The classification of hypertension in the children and adolescents was carried out taking into account the guidelines of the 2016 European Society of Hypertension (ESH) report, as presented below:

1. Normal blood pressure—value of systolic pressure and diastolic pressure below the 90th percentile for sex, age and height;
2. High normal arterial pressure (Europe)/increased blood pressure (USA)/value of systolic pressure and/or diastolic pressure between the 90th and 95th percentile for sex, age and height or pressure equal to or higher than 120/80 mm Hg
3. Hypertension—systolic and/or diastolic pressure value equal to or higher than the 95th percentile for sex, age and height in three independent measurements^17^.

### Physical activity measurement

To assess physical activity, the Actigraph accelerometer wGT3X-BT (ActiGraph, Pensacola, Florida) was used, meeting the EN60601-1 standard in the scope of safety of electrical devices in Directive 93/42 EEC.

Using the accelerometer, the number of steps per day was assessed in the examined children. The normal number of steps was defined as greater than or equal to 11,500 steps per day for children and teenagers, for both girls and boys^13^.

The device was to be worn on an elastic waist belt, above the right iliac plate from waking up until going to bed, for 7 consecutive days (only being removed for bathing and swimming). The study included data from children who had at least 3 working days with a minimum wear time of 10 h and 1 weekend day with a minimum wear time of 8 h. Non-wearing time was defined as 60 consecutive minutes without recorded movement. A 5-s time interval (epoch) and a frequency of 30 Hz were used.

### Statistical analysis

The statistical analysis of the results was carried out using the Statistica 10.1PL software. Values of basic descriptive statistics were measured together with the assessment of the significance of differences between girls and boys using the Mann–Whitney test. In the case of numerical features, the distribution characteristics of their values were presented using selected descriptive statistics.

For factors of a numerical nature, a comparison was first made of their distribution in a dichotomous division into groups defined by the classification of blood pressure.

The significance of differences between these groups in the distribution of a given factor was assessed using the Kruskal–Wallis test. For features of nominal character, the chi-square test of independence was used. The most frequently used rules regarding the value of the test probability (p) were adopted, proving the statistical significance of the relationship under consideration:

- p < 0.05 means a statistically significant relationship;
- p < 0.01 means a highly significant relationship;
- p < 0.001 means a very highly statistically significant relationship.

The study also used the multivariate analysis of variance Anova. Using the logistic regression model, the odds ratio (OR) of the effect on the probability of hypertension was estimated.

## Results

Based on the criteria of the European Society of Hypertension of 2016, subjects with normal blood pressure values, high normal pressure and arterial hypertension were defined. 61.8% of the subjects had normal blood pressure, 19% had high normal blood pressure, and 19.3% hypertension (Table 1).

**Table 1:** Classification of blood pressure in the study group.

Blood pressure classification	Number of participants	Percent
Normal pressure	619	61.8%
High normal pressure	190	19.0%
Hypertension	193	19.3%

Normal blood pressure—below the 90th percentile for sex, age and height; High normal arterial pressure—value of systolic pressure and/or diastolic pressure between the 90th and 95th percentile for sex, age and height; Hypertension—systolic and/or diastolic pressure value equal to or higher than the 95th percentile for sex, age and height.

Analysis of the results showed that in each age group the number of steps in the group with hypertension is lower compared to children with normal blood pressure. In the groups of 7–11 years and 12–15 years these differences are statistically significant. In the youngest children, the number of steps is also smaller in the group with hypertension, but this difference is not statistically significant (Table 2).

**Table 2:** The average number of steps per day and hypertension by age and sex of subjects.

Age group	Average number of steps per day [thousand]												p
	Pressure classification												
	Normal pressure				High normal pressure				Hypertension				
	N	$\overline{x}$	Me	s	N	$\overline{x}$	Me	s	N	$\overline{x}$	Me	s	
**Age**													
4–6 years	122	8.3	7.8	2.5	60	8.3	7.6	3.1	57	8.2	7.5	2.7	0.885
7–11 years	284	9.6	9.4	2.7	84	9.7	9.5	2.9	101	8.8	8.6	2.6	0.006
12–15 years	211	9.4	8.9	3.1	46	10.3	10.3	3.4	34	8.2	7.3	2.8	0.011
**Sex**													
Girls	275	9.0	8.9	2.6	96	9.0	8.6	3.1	103	8.4	7.9	2.7	0.055
Boys	342	9.4	9.0	3.0	94	9.9	9.6	3.2	89	8.7	8.4	2.5	0.017

*N* number of participants, $\overline{x}$ average, *Me* median value, *s* sample standard deviation, *p* probability level, Normal blood pressure—below the 90th percentile for sex, age and height; High normal arterial pressure—value of systolic pressure and/or diastolic pressure between the 90th and 95th percentile for sex, age and height; Hypertension—systolic and/or diastolic pressure value equal to or higher than the 95th percentile for sex, age and height.

The number of steps in boys with hypertension was significantly lower compared to those with normal blood pressure (8700 steps vs. 9400 steps). In girls, the number of steps in those with hypertension was also lower, but the difference was not statistically significant (Table 2).

A very statistically significant correlation exists between the incidence of hypertension and the meeting of the norm of recommended daily number of steps. Among the children who take at least 11,500 steps a day, approximately every ninth child (11.1%) has hypertension, while among the children with a number of steps below 11,500, there are almost twice as many (approx. 22%) (Table 3).

**Table 3:** Classification of the daily number of steps and blood pressure in children.

Pressure classification	Classification of the daily number of steps		Total
	Below norm	Norm	
Normal pressure	472 (60.9%)	147 (64.6%)	619
High normal pressure	135 (17.5%)	55(24.3%)	190
Hypertension	167 (21.6%)	26 (11.1%)	193
Total	774	228	1002

“norm”—at least 11,500 steps a day; “below norm”—a number of steps a day below 11,500.

In the entire study group, a significantly lower risk of hypertension was observed when the number of steps was normal (OR is 0.45 and the upper confidence limit for OR is 0.71). These relationships occur among both girls and boys—the strength of the dependence is slightly higher among boys (OR = 0.36 vs. 0.52 among girls). This relationship is most evident in the group of 7–11-year-old children, while slightly less among 12–15-year-olds (dependence on the border of statistical significance). There was no statistically significant relationship in the group of children 4–6 years (Table 4).

**Table 4:** Incidence of hypertension depending on the number of steps in girls and boys.

Systolic blood pressure and/or diastolic blood pressure	Classification of the daily number of steps										p
	Below norm				Norm						
	N		%		N		%		OR		
**Whole group**											
≥ 90th cc	302		39.1%		80		35.4%		0.85 (0.63–1.16)		0.318
≥ 95th cc	167		21.6%		25		11.1%		0.45 (0.29–0.71)		0.000
**Girls**											
≥ 90th cc	156		43.2%		43		38.1%		0.81 (0.52–1.24)		0.332
≥ 95th cc	87		24.1%		16		14.2%		0.52 (0.29–0.93)		0.025
**Boys**											
≥ 90th cc	146		35.4%		37		32.7%		0.89 (0.57–1.38)		0.595
≥ 95th cc	80		19.4%		9		8.0%		0.36 (0.17–0.74)		0.004
**4–6 years**											
≥ 90th cc	93	49.2%		24		48.0%		0.95 (0.51–1.78)		0.879	
≥ 95th cc	47	24.9%		10		20.0%		0.76 (0.35–1.63)		0.473	
**7–11 years**											
≥ 90th cc	162	40.1%		23		35.4%		0.82 (0.47–1.41)		0.470	
≥ 95th cc	94	23.3%		7		10.8%		0.40 (0.18–0.90)		0.023	
**12–15 years**											
≥ 90th cc	47	26.1%		33		29.7%		1.20 (0.71–2.03)		0.502	
≥ 95th cc	26	14.4%		8		7.2%		0.46 (0.20–1.06)		0.062	

*N* number of participants, *p* probability level, OR odds ratio with 95% confidence interval, 90th, 95th percentile, “*norm*” at least 11,500 steps a day; “*below norm*” a number of steps a day below 11,500.

The prevalence of hypertension in children with excess body weight is higher than in children with normal weight. However, this difference is not statistically significant. Only in the case of the oldest age group does the result approach the borderline of statistical significance (p = 0.056), and the odds ratio of hypertension in the group of overweight or obese children in relation to children with normal body weight is 2.08. In the group of children 4–6 years old with normal body weight, hypertension was diagnosed more often compared to children with excess body weight in 22.2% (Table 5).

**Table 5:** The occurrence of arterial hypertension depending on the body weight of the examined children.

Systolic blood pressure and/or diastolic blood pressure	Occurrence of overweight/obese					p
	Normal BMI		Overweight/obese			
	N	%	N	%	OR	
**All participants**						
≥ 90 cc	319	38.3%	64	38.1%	0.99 (0.70–1.39)	0.961
≥ 95 cc	156	18.7%	37	22.0%	1.23 (0.82–1.84)	0.323
**4–6 years**						
≥ 90 cc	99	48.8%	18	50.0%	1.05 (0.52–2.13)	0.892
≥ 95 cc	49	24.1%	8	22.2%	0.90 (0.38–2.10)	0.804
**7–11 years**						
≥ 90 cc	158	39.5%	27	39.7%	1.01 (0.60–1.71)	0.974
≥ 95 cc	84	21.0%	17	25.0%	1.25 (0.69–2.28)	0.459
**12–15 years**						
≥ 90 cc	62	27.0%	19	29.0.7%	1.14 (0.62–2.11)	0.665
≥ 95 cc	23	10.0%	12	18.8%	2.08 (0.97–4.45)	0.056

## Discussion

The presented study showed that children in the entire study group performed on average 9100 steps during the day. The lowest number of steps was recorded in the youngest children (8200 steps). In the groups of 7–11 years and 12–15 years it was on average 9400 steps/day. Neither sex nor age significantly differentiated the number of steps performed per day. The group of the youngest children, mostly dependent on their parents or guardians, take part in activities together with them. In the modern world, busy parents are often not able to devote enough free time to their children, who instead spend more time watching TV or playing computer games. Clear differences were noted in the number of steps depending on blood pressure. In each age group, children with hypertension take far fewer steps than those with healthy blood pressure. Boys with hypertension take an average of 8700 steps, while for those with normal blood pressure it is 9400 steps a day. Only in the youngest group of children are these differences not statistically significant.

At the level of the whole group, the risk of hypertension is clearly lower when the number of steps is normal (OR is 0.45, and the upper confidence limit for OR is 0.71). Among the children who meet the norm, i.e. take at least 11,500 steps a day, approximately every ninth child (11.1%) has hypertension, while among the children with a number of steps below 11,500 there are almost twice as many (about 22%). These relationships occur among both girls and boys—the strength of the relationship is slightly higher among boys (OR = 0.36 vs. 0.52 among girls).

Similarly, Pagels et al., in groups of children 3–5 years old in Sweden and the USA, noted a slightly lower average number of steps 7313 (± 3042) and that this increased with the children’s age^18^. In 6-year-old Japanese children, Tanaka found that the number of steps was 13,037 (± 2846) steps/day^11^. Herbert, however, showed 6-year-old Polish children to be taking 11,677 steps per day for boys, while in girls it was 9160 steps. In the same study, 7-year-old boys took 8300 steps on average, while girls took 7300 steps on average^19^. It is worth noting that these age groups represented a change from kindergarten to primary school, which may go some way to explaining the opposite trend in the number of steps as children starting primary school begin spending more time sitting at desks. In their research, Vale et al. noted that preschool children in Portugal (3–6 years old) perform an average of 9177 steps a day and may be considered to be inadequately active^20^. On the other hand, Gabel, in a study of Canadian children, suggests that a number of only 6000 steps a day for children aged 3 to 5 years meets the recommendations for physical activity^21^.

Similar results have been obtained by authors in the study of adults. Research by Iwane indicates that in adult patients with hypertension 10,000 steps a day or more, regardless of the intensity or duration of exercise, effectively lowers blood pressure, increases exercise capacity and reduces the activity of sympathetic nerves^22^. The same physical activity (10,000 steps per day) for overweight people of age 35–59 years caused systolic blood pressure (SBP) to be significantly lower − 13.74 mmHg^23^. A similar effect was obtained in patients with type 2 diabetes and hypertension^24^.

The mechanisms by which physical activity reduces blood pressure in children and adolescents are currently not identified. One of the factors that cause a beneficial effect of activity on blood pressure is nitric oxide, which is secreted under the influence of exercise. It exhibits a vasodilating effect. In addition, potassium ions are released during muscle contraction. This causes hyperaemia as well as hyperpolarization of vascular smooth muscle and endothelial cells^25^. In a study on 14-year-old children with obesity, Watts et al. showed, as a result of 8 weeks of strength training, endothelium-dependent dilatation of the brachial artery to levels observed in a group of non-obese people. After cessation of exercise, arterial reactivity returned to pre-training level within eight weeks, suggesting that vascular adaptation to exercise training is transient and remains sensitive to the negative effects of obesity if children return to lower levels of physical activity^26^. Data from Reed et al. also indicate that aerobic fitness is associated with arterial compliance in children aged 9–11 years, which confirms the concept that physical fitness can have a protective effect on the cardiovascular system^27^.

According to Torrance et al., 40-min, moderate or vigorous physical activity based on aerobics, 3–5 days a week is required to improve vascular function and reduce blood pressure in obese children^28^.

Regular physical activity results in a decrease in inflammatory markers, including proinflammatory cytokines and CRP, and a reduction in their production by adipose, muscle and endothelial cells, especially in response to moderate exercise^29^. Practicing both short and regular physical exercise allows the lowering of blood glucose levels, an increase in insulin sensitivity, reduction in the amount of fat and improvement of cardiovascular function. In their research, Andersen et al., noted that physical activity is associated with lower blood pressure and a healthier blood lipid profile in children. Muscle strength and endurance had an effect on blood lipid levels and insulin sensitivity, although the effect was less for muscle strength than for aerobic exercise^30^. As some authors suggest, adolescents should take moderate physical exercise. Intense physical activity is associated with higher systolic pressure and lower heart rate in healthy adolescents^31^. In a meta-analysis by Kelley et al., it was also found that physical activity does not leads to a significant reduction in blood pressure^32^.

Further research based on objective measurement of physical activity is required to confirm the inverse relationship between physical activity and blood pressure, especially in children and adolescents, to effectively prevent the development of hypertension. In particular, the hypothesis that volume, not intensity, of physical activity is important in regulating blood pressure should be considered.

There are some limitations to this study. First, its cross-sectional and observational nature prevents cause-and-effect inference about the relationship between physical activity and blood pressure. In addition, the study did not collect other possible influencing factors, such as diet and salt intake, related to high blood pressure that could have influenced outcomes. The relationship related to social status was also not assessed, and other types of physical activity, like swimming, were taken into account. Another limitation of the study is the lack of blood pressure measurements on two separate specimens (days) to detect persistent hypertension and avoid the error associated with a one-day blood pressure reading.

## Conclusions

Study has shown that a higher level of physical activity is undoubtedly associated with a lower risk of high blood pressure. The number of steps in the groups with hypertension is lower compared to children with normal blood pressure. In the older children (groups of 7–11 years and 12–15 years) these differences are statistically significant. In the youngest children, the number of steps is also smaller in the group with hypertension, but this difference is not statistically significant. A significantly lower risk of hypertension was observed when the number of steps was over or equal 11,500 steps per day.
